# Use of a Guinea Pig-Specific Transcriptome Array for Evaluation of Protective Immunity against Genital Chlamydial Infection following Intranasal Vaccination in Guinea Pigs

**DOI:** 10.1371/journal.pone.0114261

**Published:** 2014-12-11

**Authors:** Shradha Wali, Rishein Gupta, Ronald L. Veselenak, Yansong Li, Jieh-Juen Yu, Ashlesh K. Murthy, Andrew P. Cap, M. Neal Guentzel, James P. Chambers, Guangming Zhong, Roger G. Rank, Richard B. Pyles, Bernard P. Arulanandam

**Affiliations:** 1 South Texas Center for Emerging Infectious Diseases and Center of Excellence in Infection Genomics, University of Texas at San Antonio, One UTSA Circle, San Antonio, Texas 78249, United Stats of America; 2 Departments of Pediatrics and Microbiology & Immunology, University of Texas Medical Branch, 301 University Boulevard, Galveston, Texas 77555, United States of America; 3 US Army Institute of Surgical Research, 3650 Chambers Pass, BHT2, Building 3610/Room224-1, Fort Sam Houston, Texas 78234, United States of America; 4 Department of Pathology, Midwestern University, Downer's Grove, Illinois, 60148, United States of America; 5 Department of Microbiology and Immunology, University of Texas Health Science Center at San Antonio, 7702 Floyd Curl Drive, San Antonio, Texas 78229, United States of America; 6 Department of Microbiology and Immunology, University of Arkansas for Medical Sciences, and Arkansas Children's Hospital Research Institute, Little Rock, Arkansas 72202, United States of America; Public Health England, United Kingdom

## Abstract

Guinea pigs have been used as a second animal model to validate putative anti-chlamydial vaccine candidates tested in mice. However, the lack of guinea pig-specific reagents has limited the utility of this animal model in *Chlamydia sp*. vaccine studies. Using a novel guinea pig-specific transcriptome array, we determined correlates of protection in guinea pigs vaccinated with *Chlamydia caviae* (*C. caviae*) via the intranasal route, previously reported by us and others to provide robust antigen specific immunity against subsequent intravaginal challenge. *C. caviae* vaccinated guinea pigs resolved genital infection by day 3 post challenge. In contrast, mock vaccinated animals continued to shed viable *Chlamydia* up to day 18 post challenge. Importantly, at day 80 post challenge, vaccinated guinea pigs experienced significantly reduced genital pathology - a sequelae of genital chlamydial infections, in comparison to mock vaccinated guinea pigs. Sera from vaccinated guinea pigs displayed antigen specific IgG responses and increased IgG1 and IgG2 titers capable of neutralizing GPIC *in vitro*. Th1-cellular/inflammatory immune genes and Th2-humoral associated genes were also found to be elevated in vaccinated guinea pigs at day 3 post-challenge and correlated with early clearance of the bacterium. Overall, this study provides the first evidence of guinea pig-specific genes involved in anti-chlamydial vaccination and illustrates the enhancement of the utility of this animal model in chlamydial pathogenesis.

## Introduction

Diagnosis and treatment of *Chlamydia trachomatis*, the leading cause of bacterial sexually transmitted infections globally, contributes significantly to increasing health-care costs [1], [2]. Despite decades of epidemiological studies [3], [4], [5], sexual health programs [6], improved diagnosis [7], [8], [9] and antibiotic treatment regimens [10], increasing incidence rates are a cause for concern. A licensed anti-chlamydial vaccine might prove to be efficacious in controlling genital infections. To this end, our laboratory [11], [12], [13], [14], [15] and others [16], [17], [18], [19], [20], [21] have reported on vaccination strategies against genital *C. trachomatis*. We have established that intranasal (i.n.) vaccination with recombinant (r) chlamydial protease/proteasome-like activity factor (rCPAF; derived from *C. trachomatis* serovar L2) significantly abrogated/reduced intravaginal (i.vag.) *C. muridarum* infection [11], upper genital tract genital pathology [12] and infertility in mice [22]. rCPAF vaccination induced protective immunity *via* induction of IFN-γ producing Th1-type antigen-specific CD4^+^ T cells [12], [13]. However, due to differences in immunological responses [23], [24], [25], [26], and chlamydial strain susceptibilities between mice and humans [25], [27], the bench to bedside transition for an anti-chlamydial vaccine has not been achieved [28], [29].

Several translational animal models, including rhesus and grivet monkeys, pig-tailed macaques, marmosets, pigs and guinea pigs have been reported for the study of chlamydial genital infection [24], [26], [27], [30], [31]. Although no animal model is ideal, the guinea pig model offers distinct advantages [26], [31]. Specifically, the causative agent of guinea pig inclusion conjunctivitis (GPIC or *C. caviae*), produces a genital tract infection remarkably similar to human *C. trachomatis* genital infection with regard to pathogenesis, immunity, and the ability to be transmitted sexually [26], [32]. Also, *C. caviae* is a natural pathogen of the guinea pig and is able to infect superficial epithelial cells in the ectocervix and endocervix [33], and to produce ascending infection to the endometrium and oviducts [32]. The female reproductive system of the guinea pig is closely related to the human with regard to histological features and physiology. The guinea pig has a 15–17 day estrous cycle that includes active hormone secretion from a corpus luteum, eliminating the need for hormonal pre-conditioning necessary for infection, colonization and pathogen ascension in other animal models [26], [32]. However, the lack of guinea pig specific reagents has limited the use of this animal model for evaluating the efficacy of putative vaccine candidates.

In the current study, we determined protective immunity against i.vag. infection in guinea pigs vaccinated with chlamydial elementary bodies (EBs) - known to provide robust protection against genital challenge(s) [34], [35]. Specifically, we immunized female guinea pigs intransally (i.n.) with *C. caviae* EBs, or delivered PBS to controls (mock vaccinated). The animals were then challenged i.vag. with *C. caviae*. Utilizing a novel guinea pig innate and adaptive immunity-associated gene qPCR array for transcriptome analysis, we observed the regulation of genes that may contribute to innate responses, Th1-cellular/inflammatory, and Th2-humoral immunity. These analyses revealed that Th-1 and Th-2 associated gene expression was increased in *C. caviae* EB vaccinated guinea pigs by day 3 post challenge. Importantly, *C. caviae* EB vaccinated guinea pigs cleared i.vag. infection by day 3 post challenge and displayed significantly less upper genital pathological damage compared to mock vaccinated guinea pigs.

## Materials And Methods

### Bacteria

Chlamydial stocks (obtained from Dr. Harlan Caldwell at the Rocky Mountain Laboratory, NIAID/NIH) were prepared as described previously [36]. EB (infectious form) of *C. caviae* were harvested from infected HeLa cells and stored at −80°C in sucrose–phosphate-glutamine (SPG) buffer. *C. caviae* stock titers were determined [11] and diluted appropriately in PBS for both i.n. immunization and i.vag. challenge.

### Guinea Pigs

Dunkin Hartley strain guinea pigs (350–450 g) were purchased from Charles River Laboratories (Massachusetts, USA) and were housed in the AAALAC-accredited University of Texas at San Antonio Vivarium. Food and water were supplied *ad libitum* and all experimental studies were completed humanely and followed the recommendations in the Guide for the Care and Use of Laboratory Animals of the National Institutes of Health. The protocol (IS0146) was approved by the Institutional Animal Care and Use Committee (IACUC) of the University of Texas at San Antonio.

### Immunization and Challenge

Guinea pigs were immunized i.n. with 1×10^5^ EB *C. caviae*. Immunized guinea pigs were rested for one month and then challenged i.vag. with 1×10^5^ EB of *C. caviae*. Following challenge, guinea pigs were swabbed every 3 days and the swabs were used to infect HeLa cell monolayers to determine infection status. Chlamydial inclusions were detected at 30 h using an anti-*Chlamydia* genus specific rabbit monoclonal primary antibody and goat anti-rabbit IgG secondary antibody conjugated to FITC plus Hoescht nuclear stain.

### Anti-*Chlamydia* Antibody Titers

Guinea pigs were bled from the lateral saphenous leg vein to produce sera pre-vaccination (day 0) and post-challenge (day 15) as described previously [37]. Microtiter plates were coated with 1×10^5^ EB of UV-inactivated *C. caviae* and incubated overnight at 4°C. Each serum sample was diluted serially and incubated for 2 h, followed by incubation with goat anti-guinea pig total IgG or anti-IgG1 or anti-IgG2 conjugated to horseradish peroxidase (HRP). Tetramethylbenzidine substrate was added and the absorbance quantified at 630 nm using a µQuant ELISA plate reader (BioTek Instruments, Winooski, VT). Reciprocal antibody titers were calculated for each group of guinea pigs using 50% maximal binding of serum.

### Quantification of Neutralizing Antibody Titer

Sera from each guinea pig, post-immunization (day 30) and after challenge (day 15), were heat-treated (56°C, 30 min) or untreated and diluted 1∶25 in DMEM before plating into wells of a 96 well microplate. Sera from each animal was incubated with 2×10^4^ EB of *C. caviae* for 1 h at 37°C. Following incubation, the surviving infectious bacteria in each well were transferred to a 24 well plate containing 80% confluent HeLa cell monolayers. Infection proceeded for 30 h and infectivity was determined using a fluorescent microscope to measure bacterial inclusions formed inside HeLa cells as described above. Neutralization and percentage reduction in chlamydial EB were calculated by comparing the change in infectivity between vaccinated and mock-vaccinated samples.

### Guinea Pig Transcriptome Analysis

**RT-PCR Array Development**:. The novel guinea pig array was developed using the genomic sequence for the guinea pig (*Cavia porcellus*) available through the Ensemble database (http://www.ensembl.org/Cavia_porcellus/Info/Index). An optimized primer design algorithm and associated thermocycling program allowed for 96 distinct targets to be arrayed on one 96 well plate (92 targets + 4 housekeeping gene controls) (S2 Figure). The PCR cycling parameters, completed in either CFX or CFX Connect Real-Time PCR detection systems (Bio-Rad), were 8 cycles of 95°C for 30 seconds, 48°C for 30 seconds, and 72°C for 30 seconds followed by a 40 cycle amplification of 95°C for 15 seconds, 56°C for 20 seconds and 72°C for 20 seconds during which real time data were acquired at the annealing step. The four housekeeping genes were beta actin, eukaryotic elongation factor 1 alpha (eEF1a1), glyceraldehyde 3-phosphate dehydrogenase (GAPDH), hypoxanthine-guanine phosphoribosyltransferase 1 (HPRT1). Expression of these genes across tissues and conditions was found to be statistically consistent by correlation analysis (R^2^ = 1.0).

The assembled quantitative RT-PCR (qRT-PCR) array targeted guinea pig genes encoding innate and adaptive immunity components essential to efficacious vaccination and infection clearance, allowing specific pathways involved in the vaccination response to be identified. Screening with the array allowed for subsequent targeted qRT-PCRs to be performed on differentially regulated genes of interest. Designed primers were first validated by RT-PCR of cDNA from PMA/ionomycin-treated and mock treated guinea pig splenocytes; primer pairs were selected if they produced a single product (confirmed by melting temperature and agarose gel electrophoresis) with amplification efficiencies >80%. The primer sequences for genes on the array were same as described elsewhere [38]. The assembled array was validated with a series of control samples including individual technical replicates of splenocytes and negative controls including no DNA (water only), RNA and unrelated (human) cDNA. Array specificity was further validated using cDNA created from approximately 200 distinct outbred Swiss Hartley guinea pigs from at least two different colonies with no obvious failures in target amplification. Finally, high resolution melting temperature (Tm) analysis of each PCR product confirmed that amplification represented the proper genetic target.

**Preparation of Guinea Pig Genital Tract Nucleic Acids**:. RNA was extracted from genital tract tissues harvested from three humanely euthanized guinea pigs from each group at the indicated times post challenge using the Aurum RNA extraction system (Bio-Rad; Hercules CA). Briefly, small (<3 mm^3^) tissue pieces representing the lower (vagina and cervix) and upper (uterine horns and oviducts) genital tracts of individual guinea pigs were homogenized in Aurum lysis solution supplemented with 1% beta-mercaptoethanol. Following the kit instructions, total RNA (∼2 ug per sample) was collected in the 96 well format and then immediately converted into cDNA using the iScript cDNA synthesis kit (Bio-Rad). The resulting cDNA was analyzed by PCR array (∼2 ng of RNA per well) immediately or stored at −20°C until analysis. Gene expression data were normalized using quantile transformation to provide a more uniform distribution of intensities as described by Bolstad *et al*. [39]. This approach normalized each gene expression level and each sample to the others to account for differences in RNA quality and quantity. Comparisons of the transcription profiles among the lower and upper genital tracts of naïve, mock vaccinated but challenged, and vaccinated and challenged animals at days 3 and 9 post-challenge were performed using delta delta Cq analyses [40] to establish fold change (FC). The FC values were subsequently evaluated by Student's t-test (Prism v6.0; GraphPad) to identify significantly differentially regulated genes.

Selected genes that were expressed differently between groups were subsequently analyzed by qRT-PCR with single target assessment under optimal conditions established for each specific target. For these studies the same RNA used in the array was subjected to single target confirmation. Single target expression data were normalized against the averaged housekeeper expression levels for HPRT1 and eEF1a1. Expression profiles for these two housekeepers were indistinguishable across all the samples in the study (correlation coefficient R^2^ = 1.0). This approach confirmed the data from the quantile normalized Cq values generated by the array and provided accurate quantified outcomes for FC calculations. For each qPCR run, a 10-fold dilution series of known copy number was processed in parallel as described previously [41] to provide a means of extrapolation of Cq value to actual copy number in a given sample. All PCR analyses were completed in CFX real time instruments (Bio-Rad) using optimized thermocycling conditions.

### Determination of *C. caviae* Loads in Infected Genital Tracts

DNA was collected from each tissue [42], and subjected to qPCR assays. Primers targeting the tryptophan synthase gene (beta subunit) of *C. caviae* were used to quantify bacterial load in lower and upper genital tract of infected guinea pigs. A melting temperature analysis was performed to identify and confirm all qPCR products with a resulting 84°C Tm for the *C. caviae* product. Guinea pig GAPDH served as a DNA quality and quantity indicator and was used to normalize the *C. caviae* results. Guinea pig GAPDH qPCR utilized forward (5′-AAT GGG AAG CTC ACA GGT ATG G-3′) and reverse (5′-ATG TCA TCG TAT TTG GCC GGT-3′) primers and a TET-labeled TaqMan probe (5′-TET-TCC AGG CGG CAG GTC AGA TCC ACA-BHQ1-3′). The lower limit of detection for the assays was 50 copies.

### Genital Tract Pathology

Genital tract tissues of all animals harvested on day 80 post-challenge were fixed in 10% formalin, embedded in paraffin, sectioned, and stained with hematoxylin-eosin (H&E). Histological images were recorded at ×200 or ×400 magnifications under an Olympus AX80 light microscope (Olympus, Center Valley, PA) and evaluated in a blinded method for pathological damage. The microscopic findings were either graded as none/minimal (0), slight (1), moderate (2), or severe (3) histological alteration. Histological scores were obtained by examining 5 consecutive sections (2 mm-interval) of cervix, oviducts, and uterus from every animal. Scores assigned to individual guinea pigs were used to calculate the pathology scores for each group of animals and presented as mean ± standard deviation.

## Results

### Intranasally Vaccinated Guinea Pigs are Protected Against Intravaginal *C. caviae* Infection

**Figure 1:**
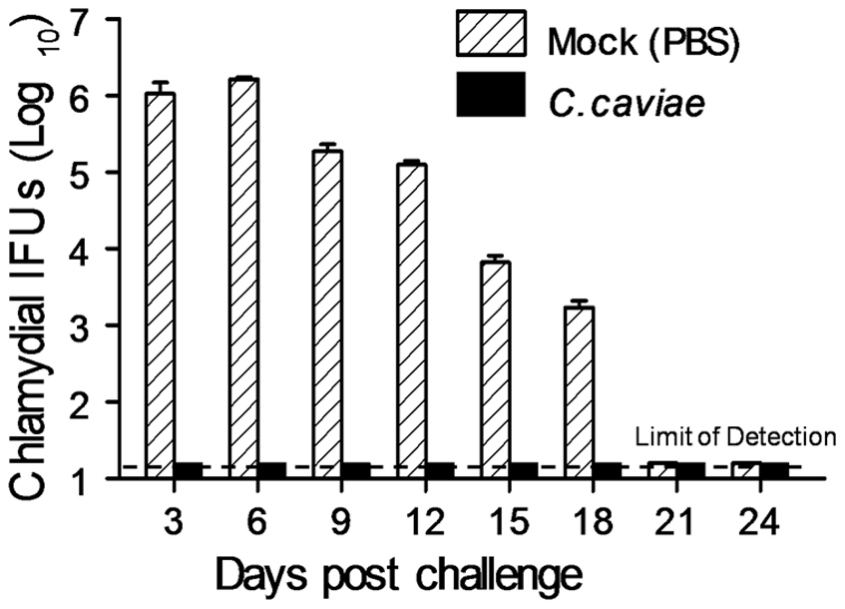
Vaccination of Guinea pigs with *C. caviae* Protects Against Genital Chlamydial Infection. Groups (5 per group) of guinea pigs were immunized i.n. with 1×10^5^ IFU *C. caviae* or treated with PBS as mock vaccination controls. All animals were rested for 30 days and challenged i.vag. with 1×10^5^ IFU *C. caviae*. Chlamydial shedding was monitored every third day after challenge for a month, and are presented as mean ± SD for each group at each time point.

Intranasal vaccination of mice with *C. muridarum* EBs has been shown to provide robust protection against genital *C. muridarum* infection [11], [12]. To establish a similar vaccination regimen against i.vag. *C. caviae* infection in guinea pigs, we i.n. immunized guinea pigs with 1×10^5^ *C. caviae* EBs. Guinea pigs administrated PBS i.n. were used as a mock vaccination control, similar to studies in mice previously reported to be comparable to an adjuvant-alone control group [15]. All guinea pigs were rested for 30 days and i.vag. challenged with 1×10^5^ *C. caviae* EBs. As shown in Fig. 1, *C. caviae* EB vaccinated animals cleared the infection at day 3 post challenge whereas mock vaccinated guinea pigs shed *C. caviae* (1×10^6^ inclusion forming units; IFU) for 6 days post challenge, followed by reduced bacterial loads from days 9–18, and no recoverable bacteria by day 21 post challenge.

### Vaccination Induced *C. caviae* Neutralizing Antibody Production in Guinea Pigs

**Figure 2:**
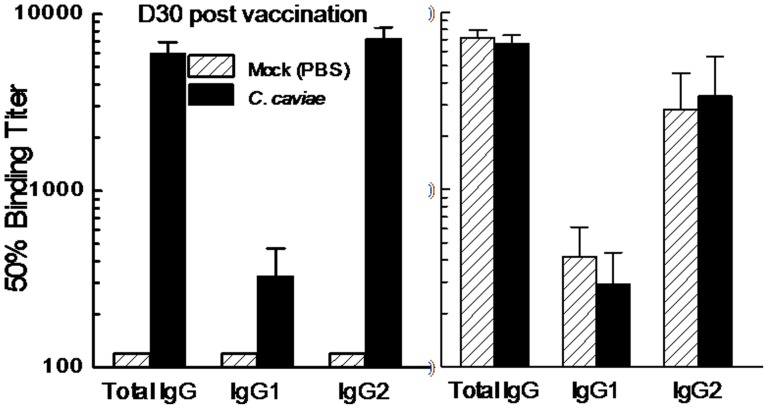
Intranasal Vaccination with *C. caviae* Induced Robust Humoral Responses. Groups (5 per group) of guinea pigs were immunized i.n. with 1×10^5^ IFU *C. caviae* or treated with PBS as mock vaccination controls. All animals were rested for 30 days and challenged i.vag. with 1×10^5^ IFU *C. caviae*. Serum antibody titer (total antibody, IgG1 and IgG2a) to UV-inactivated *C. caviae* was determined at day 30 post vaccination (A) or at day 15 after challenge (B). Total Ig, IgG1 and IgG2a antibody titers were significantly (*p*<0.05) elevated in *C. caviae* EB vaccinated guinea pigs compared to mock vaccinated animals prior to challenge (A), but not after challenge (B).

**Figure 3:**
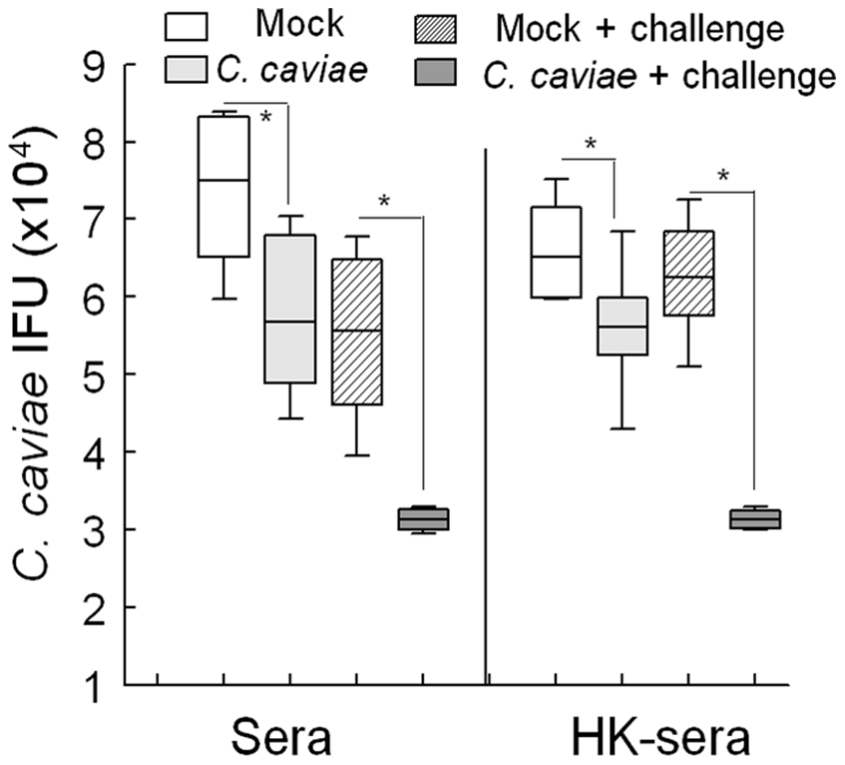
*C. caviae* Immune Serum and Chlamydial Infectivity. Sera obtained from guinea pigs (n = 5 per group) 30 days post vaccination with *C. caviae* or mock (PBS) or 15 days after challenge (+ challenge) were heat-inactivated (HK) at 56°C for 30 min or left untreated and diluted 1∶25 in DMEM before addition into 96 well plate. Sera from each animal was incubated with *C. caviae* (2×10^4^ IFU) for 1 hr in a shaking incubator, then sera/bacterial mixtures were added to HeLa cells (0.01 MOI) for bacterial enumeration. Bacterial numbers for each group were presented as a box-and-whisker plot. * *p*<0.05.

Vaccination with *C. caviae* EB produced elevated levels of total *Chlamydia*-binding IgG, compared to mock vaccinated guinea pigs at day 30 post-vaccination (Fig. 2A). However, anti-*C. caviae* serum titers were comparable in *C. caviae* and mock vaccinated guinea pigs at 15 days post challenge (Fig. 2B). Given that a significant difference in bacterial shedding between vaccinated and mock vaccinated animals was observed (Fig. 1), we next compared the neutralization capacity of sera collected from both groups of guinea pigs. As shown in Fig. 3, sera collected day 30 post vaccination from *C. caviae* EB vaccinated guinea pigs reduced infectivity in HeLa cells by approximately two logs compared to sera from mock vaccinated guinea pigs. This difference in ability to neutralize *C. caviae in vitro* increased to approximately three logs at day 15 post-challenge, supporting the conclusion that sera from *C. caviae* EB vaccinated guinea pigs generated antibodies capable of neutralizing *C. caviae* better than mock immunized animals. Because similar trends were observed in heat-treated sera we conclude that the neutralizing effects observed were independent of complement.

### *C. caviae* EB Vaccinated Guinea Pigs are Protected Against Development of Reproductive Tract Pathology Following Intravaginal Challenge

**Figure 4:**
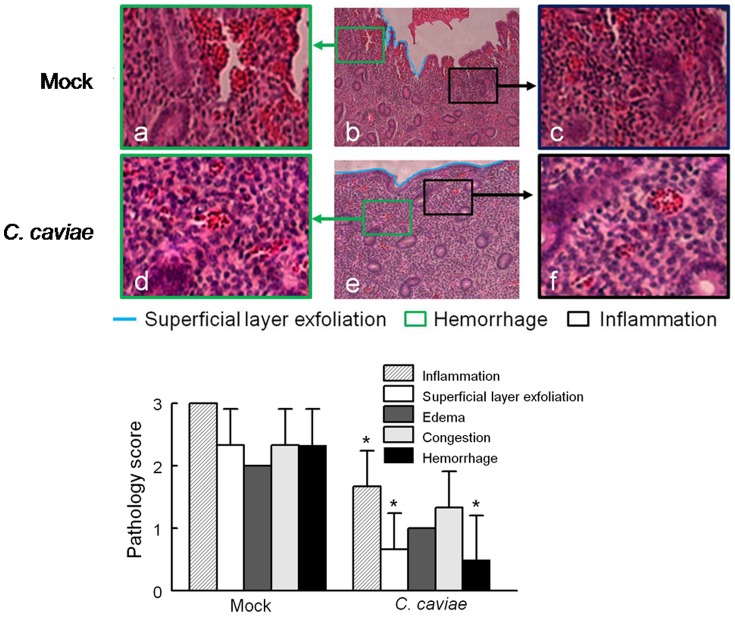
Effect of *C. caviae* Vaccination on Histopathological Lesions in the Genital tract from Guinea pigs following Chlamydial Challenge. The genital tracts from each humanely euthanized guinea pig were removed at day 80 post *C. caviae* challenge fixed and embedded and then sectioned, and analyzed microscopically after H&E staining. (A) Representative photomicrographs of histological sections from uterine tissues are shown for each group of challenged guinea pigs with *C. caviae* EB vaccination (EB, n = 3) or mock vaccinated (Mock, n = 3). The superimposed images are magnifications of the regions of the indicated boxes to show details of inflammatory cell infiltration (c and f) and hemorrhage (a and d). Original magnification of the images (b and e) is ×200, while a, c, d and f are ×400. The light blue dash lines mark superficial layer exfoliation of the endometrial epithelium of the uterus (b), whereas the light blue solid lines indicate the intact endometrial epithelium of the uterus (e). Histopathological injury scores were calculated from five distinct morphological parameters (inflammatory cell infiltration, superficial layer exfoliation, edema, congestion and hemorrhage) in the uterus (B). Scores were calculated by examination of 5 consecutive sections (2 mm-interval) in every animal. Graphs expressed as mean ± SD, and compared using paired *t*- test. The asterisk indicates statistically significant differences (* *p*<0.05) between the *C. caviae* group and the mock group for the respective parameter.

To evaluate the effect of *C. caviae* EB vaccination on development of pathological lesions in the genital tract, sections were obtained from challenged guinea pigs at day 80. Previous extensive analyses have demonstrated the suitability of this time-period to evaluate the upper genital tract sequelae following i.vag. *Chlamydia* challenge [12], [15], [43]. Histological analysis of the uterus 80 days after chlamydial challenge of mock-vaccinated animals revealed pathological damage that was characterized by the presence of a severe inflammatory cell infiltration (majority of the inflammatory cells were lymphocytes and macrophages) (Fig. 4A), moderate superficial layer exfoliation (Fig. 4A) and hemorrhage (Fig. 4A, a and b). In contrast, *C. caviae* EB vaccinated animals had an intact endometrial epithelium (Fig. 4A, e), reduced inflammation (Fig.4A, e and f) and hemorrhage in the uterus (Fig.4A, e and d). The mean histopathology severity scores for the uterus demonstrated significantly (*p*<0.05) reduced inflammatory cell infiltration, superficial layer exfoliation, and hemorrhage upon *C. caviae* EB vaccination compared to controls (Fig. 3B). Congestion and edema were reduced in vaccinated animals, but these scores were not statistically different from the mock-vaccinated guinea pigs.

Histopathological examination of the cervix indicated that *C. caviae* EB vaccination significantly (*p*<0.05) reduced edema compared to mock vaccinated controls (S1 Figure). Although not significant, decreased inflammatory cell infiltration (cervix and oviducts), superficial layer exfoliation (cervix), edema (oviducts) and congestion (cervix and oviducts) were observed in *C. caviae* EB vaccinated guinea pigs compared to controls (S1 Figure).

### Host Responses are Increased Following *C. caviae* EB Vaccination in Guinea Pigs

**Figure 5:**
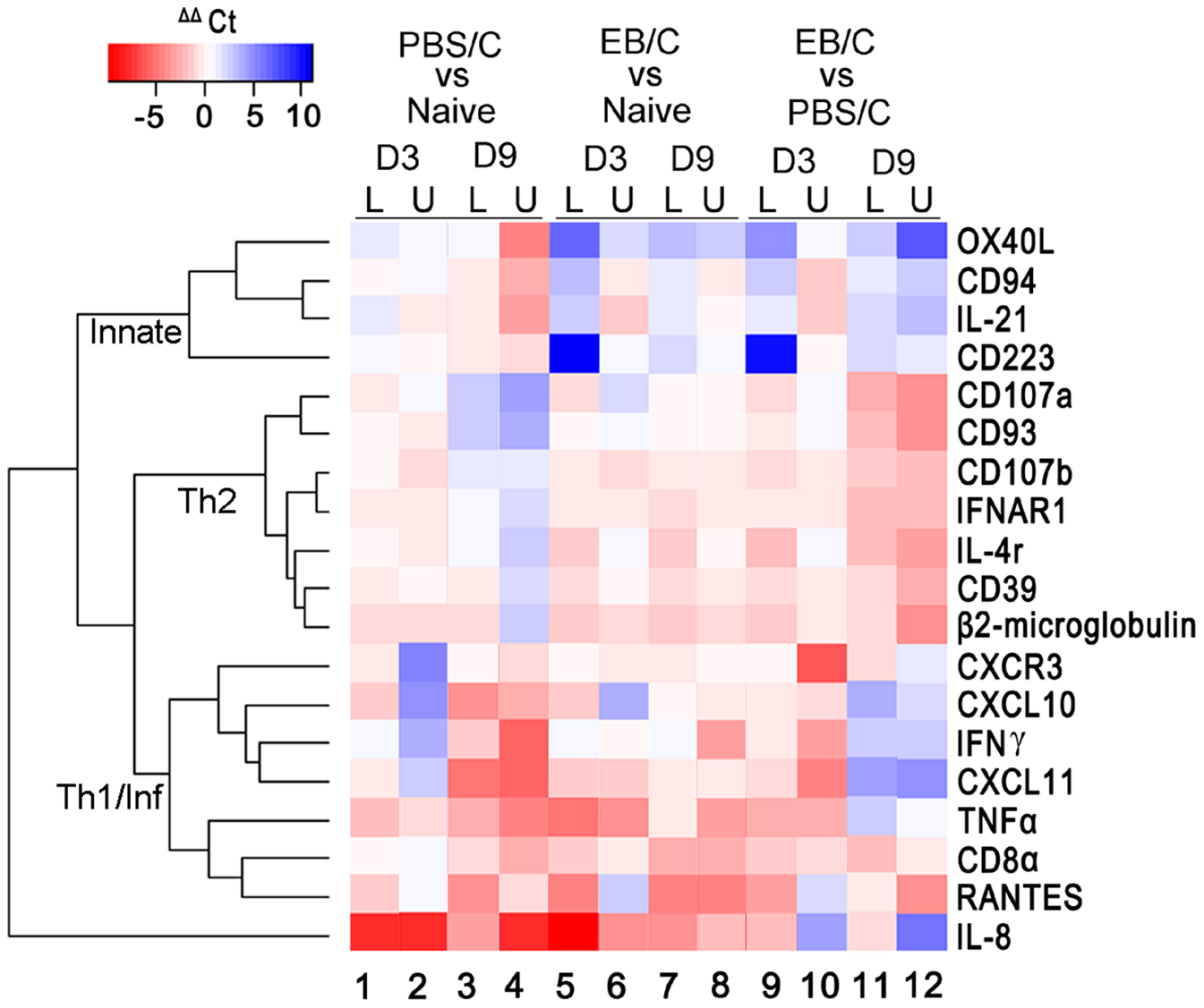
Comparative Heatmap Depiction of Differential Gene Expression using RT-PCR array screening. Three groups of guinea pigs were used for the comparative study: non vaccinated and non challenged (naïve), mock vaccinated but challenged (PBS/C), and *C. caviae* EB vaccinated and challenged (EB/C) groups. Each group contained three animals. The tissues (upper and lower genital tracts, U and L, respectively) from the respective groups of animals were collected at days 3 and 9 after challenge. Red shading indicates an increase in expression, while blue shading indicates suppression of expression, of the gene indicated on the right side of the panel. Lighter shades including white indicate similar levels of expression. Functional gene clustering is indicated by the brackets on the left showing 3 major groups consisting of innate, Th2 and Th1/inflammatory related genes.

We developed a guinea pig-specific array (S2 Figure), to screen for differences in selected immune response-related genes involved in *C. caviae* EB vaccination-induced protection (Fig. 1). Results of the qRT-PCR analyses revealed modulation of 19 highly regulated genes that were subjected to hierarchical clustering analyses for probable co-regulation of immune components in *C. caviae* EB vaccinated or mock vaccinated guinea pigs (Fig. 5). The co-regulation of innate (NK), Th2-humoral (including CD93, CD39, IL-4R, β2-microglobulin) and Th1-cellular/inflammatory responses was evident in the heat map as clustering into 3 major groups. Overall, following *C. caviae* infection (Fig. 5, lanes 1–4) NK activation genes (CD94, IL-21, and CD233) were upregulated. In contrast, Th2 humoral response-related genes were down-regulated and correlated to an increased Th1 cellular and inflammatory response by day 9.

Interestingly, when comparing *C. caviae* EB vaccinated with mock vaccinated animals after chlamydial challenge (Fig. 5, lanes 9–12), we observed increased Th1 and Th2 immune responses in vaccinated guinea pigs at day 3, with concomitant humoral response remaining enhanced at day 9 post challenge. Robust immune responses on day 3 post challenge (Fig. 5) correlated with early bacterial shedding in *C. caviae* EB vaccinated guinea pigs (Fig. 1) Next, to confirm the results of the array, single qPCR assays were performed for selected genes associated with both innate and acquired immunity. The data are summarized in Tables 1 (lower genital tract) and 2 (upper genital tract) and provided an overall confirmation of the array results (Fig. 5). The data indicated that *C. caviae* EB vaccinated animals had significantly increased expression of CXCL10, CXCL11, CD107a, CD107b, IFNαR1 and β2-microglobulin compared to controls in the lower genital tract at day 3 post challenge. Additionally, the diminished need for elevated cellular response (s) at day 9 post challenge in *C. caviae* EB vaccinated animals (bacterial clearance was observed by day 3, Fig. 1) was consistent with significantly lower expression of inflammatory- and T cell-associated genes, including CXCL10, CXCR3, CD8α, IL-21, RANTES, OX40L and IFN-γ, relative to mock vaccination (Table 1).

**Table 1:** Fold-change between groups from qPCR assays of selected immune response genes from the guinea pig lower genital tract.

a Comparison Between Groups		CXCL10	CXCL11	CXCR3	CD8α	CD107a	CD107b	IL-21	IFNAR1	RANTES	β2 µglobulin	OX40L	IFN-γ
D3	PBS/C vs Naive	6.3 ↑	3.3* ↑	1.4 ↑	1.6 ↑	2.3 ↑	1.6 ↑	1.3 ↓	1.6 ↑	1.7 ↑	2.9* ↑	1.2 ↑	1.4 ↑
	EB/C vs Naive	9.5* ↑	6.7** ↑	2.5 ↓	1.1 ↑	5.6* ↑	3.0* ↑	>10 ↓	6.1* ↑	5.6 ↑	6.8** ↑	>10 ↓	5.0 ↓
	EB/C vs PBS/C	1.4 ↑	2.0** ↑	3.5 ↓	1.4 ↓	2.5 ↑	2.0 ↑	6.0 ↓	3.3 ↑	3.3 ↑	2.5** ↑	>10 ↓	6.7 ↓
D9	PBS/C vs Naive	>10* ↑	>10 ↑	1.0 ↑	1.6 ↑	1.7 ↓	1.4 ↓	1.1 ↓	2.5 ↓	7.4* ↑	1.3 ↑	1.0 ↓	2.8 ↑
	EB/C vs Naive	1.7 ↑	1.0 ↑	5.0 ↓	1.4 ↓	1.2 ↑	1.7 ↑	5.0 ↓	3.1** ↑	1.7 ↑	2.4** ↑	>10 ↓	>10 ↓
	EB/C vs PBS/C	9.5* ↓	>10 ↓	4.2* ↓	2.3* ↓	2.0** ↑	2.5** ↑	4.8* ↓	>10** ↑	4.5* ↓	1.7** ↑	>10* ↓	>10* ↓

Arrows indicate direction of fold change.

* indicates *p*<0.05; ** indicates *p*<0.01.

a PBS/C: mock-vaccinated and challenged, EB/C: EB-vaccinated and challenged, Naïve: non-vaccinated and not challenged.

The upper genital tract analyses (Table 2) showed reduced immune gene regulation compared to lower genital tract at day 3. At day 9 post challenge, gene expression of IFN-γ and chemokines (e.g. CXCL10, CXCL11) in mock vaccinated guinea pigs, compared to *C. caviae* EB vaccination or naïve guinea pigs, correlated with active/ongoing infection (Fig. 1). Noticeably, lysosomal-associated membrane proteins, CD107a (LAMP1) and CD107b (LAMP2) were significantly downregulated at day 9 post-challenge (PBS/C vs Naïve); whereas their expression level was significantly increased in *C. caviae* EB vaccination (EB/C) compared to PBS/C animals. Lysosomal repair (accompanied with appearance of LAMP1) retains *Chlamydia* within the surviving host cell [44], but the mechanism(s) of vaccination induced protection potentially mediated by CD107 remains to be elucidated. Overall, gene expression profile(s) in *C. caviae* EB vaccinated guinea pigs correlated with reduced pathology histologically (Fig. 4) and increased immune responses to result in reduced genomic titers of *C. caviae*.

**Table 2:** Fold-change between groups from qPCR assays of selected immune response genes from the guinea pig upper genital tract.

a Comparison Between Groups		CXCL10	CXCL11	CXCR3	CD8α	CD107a	CD107b	IL-21	IFNAR1	RANTES	β2 µglobulin	OX40L	IFN-γ
D3	PBS/C vs Naive	1.1 ↓	3.3 ↓	3.3 ↓	1.4 ↓	1.2 ↑	2.3 ↑	1.4 ↓	2.1 ↑	1.4 ↑	2.3 ↑	1.3 ↓	1.1↓
	EB/C vs Naive	1.2 ↑	1.1 ↓	2.0 ↓	1.3 ↑	3.3** ↓	1.4 ↓	1.7 ↑	1.5 ↑	3.3 ↓	1.3 ↓	2.0 ↓	1.9 ↑
	EB/C vs PBS/C	1.3 ↑	3.3 ↑	1.7 ↑	2.0 ↑	4.5** ↓	3.2 ↓	2.5 ↑	1.4 ↓	4.4* ↓	3.0* ↓	1.5 ↓	2.0 ↑
D9	PBS/C vs Naive	>10 ↑	>10 ↑	2.0 ↑	2.8 ↑	>10* ↓	>10* ↓	5.2 ↑	>10** ↓	1.2 ↑	5.0 ↓	3.9* ↑	>10 ↑
	EB/C vs Naive	4.2 ↑	7.0 ↑	3.4 ↑	5.4 ↑	1.9* ↑	3.5** ↑	5.4 ↑	5.9** ↑	5.9* ↑	3.8** ↑	4.4 ↑	3.9 ↑
	EB/C vs PBS/C	2.5 ↓	7.1 ↓	1.7 ↑	2.0 ↑	>10** ↑	>10** ↑	1.0 ↑	>10** ↑	5.0 ↑	>10** ↑	1.1 ↑	2.8 ↓

Arrows indicate direction of fold change.

* indicates *p*<0.05; ** indicates *p*<0.01.

a PBS/C: mock-vaccinated and challenged, EB/C: EB-vaccinated and challenged, Naïve: non-vaccinated and not challenged.

### Evaluation of *C. caviae* Bacterial Burden by Genomic Analysis

**Figure 6:**
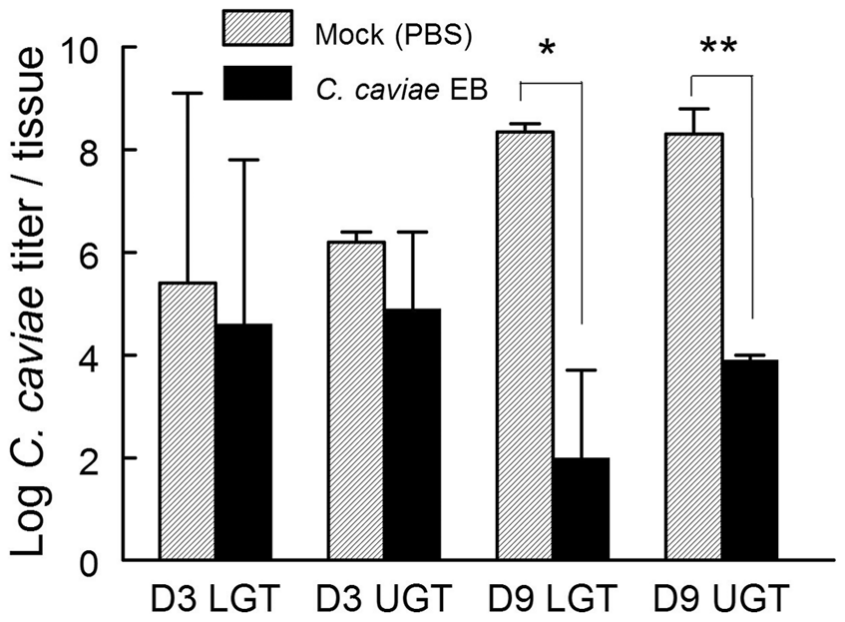
Quantitative PCR Assessment of Bacterial Genomic Burdens in Lower and Upper Genital Tracts from *C. caviae* Mock or (PBS) Vaccinated Guinea Pigs. Groups of three animals were euthanized on days 3 and 9 after *C. caviae* i.vag. challenge and tissues representing the lower or upper genital tract were aseptically collected. DNA was subjected to qPCR for GAPDH (host target used for normalization) and the single copy *C. caviae* tryptophan synthase gene (quantification of bacterial load). The average bacterial burdens for each tissue are depicted as grey (mock-vaccinated) or black (vaccinated) bars for each tissue and time point. LGT: lower genital tract. UGT: upper genital tract. * *p*<0.05, ** *p*<0.01.

In order to add to the bacterial shedding profiles of *C. caviae* EB and mock vaccinated animals (Fig. 1), bacterial burdens in lower and upper genital tract of guinea pigs at days 3 and 9 post challenge were estimated using qRT-PCR. Although *C. caviae* loads were comparable at day 3 post challenge in the lower and upper tract of vaccinated and mock-vaccinated guinea pigs (Fig. 6), by day 9, *C. caviae* EB vaccinated guinea pigs displayed 4–6 logs fewer bacterial genomes in lower and upper genital tracts. Additionally, of the twelve tissues from *C. caviae* EB vaccinated animals, three had no detectable bacterial genomes. Taken together, bacterial burdens (Fig. 6) and shedding data (Fig. 1) indicated a robust protection and generation of immune responses (Fig. 5, Table 1 and 2), were induced in *C. caviae* EB vaccinated guinea pigs compared to mock vaccination.

## Discussion

This study provides evidence that intranasal immunization with *C. caviae* provides robust protection against i.vag. *C. caviae* challenge by induction of neutralizing antibodies and induction of localized T and B cell responses within the genital compartment in the guinea pig model. Biologically, immunization reduced reproductive tract pathological sequelae that can be associated with human vaginal infections leading to impacts on fertility and susceptibility to other infections including HIV. Importantly, our study is the first to carefully interrogate the gene expression patterns in immunized animals after challenge with this extremely common bacterial STI.

The utilization of guinea pigs as an alternate and complimentary animal model to mice is highlighted by (1), the unavailability of a licensed anti-chlamydial vaccine despite several vaccine studies in mice models [11], [12], [13], [16], [19], [45], [46]; (2), cost effectiveness compared to non-human primates and bovine models [24], [30], [47], [48]; (3), remarkable similarity to human *C. trachomatis* infection with regards to bacterial ascension, colonization and related pathogenesis [32], [49], [50]; and, (4), the ability to study transmission dynamics of chlamydial infections following sex between males and females [51]. However, in spite of being one of the oldest animal models used for immunological studies [52], and in research related to several pathogens including *Chlamydia sp*. [26], [31], *Mycobacterium sp*. [53], [54], *Legionella sp*. [55], *Francisella sp*. [56] and, *Neisseria sp*. [57], the limited availability of guinea pig-specific reagents [52] has led to it being an animal model of only partial utility. The application of a novel guinea pig gene expression qRT-PCR array both advances the utility of the animal model and helps to increase our understanding of the immune outcomes of i.n. vaccination against *Chlamydia*.

Previous vaccination studies against genital *C. caviae* infection in guinea pigs have utilized oral [34], subcutaneous [17], [35], intraperitoneal [58] and i.vag. routes [59] for delivery of protective peptides [17], [58], [60] or whole EBs [34], [35]. We have extensively characterized the use of i.n. vaccination approaches to generate robust protection against genital infections in murine models [11], [12], [22]. Specifically, i.n. *C. caviae* EB immunization protected guinea pigs against i.vag. challenge (Fig. 1) and development of upper reproductive tract pathology (Fig. 4). Bacterial shedding data (Fig. 1) following i.n vaccination was found to be consistent with a previous report on whole EB subcutaneous vaccination and clearance of *C. caviae* intravaginal infection 3–6 days post i.vag. challenge [34]. Following vaccination, elevated levels of anti-*Chlamydia* IgG were detected in sera from vaccinated animals relative to naïve animals. Furthermore, engendered antibodies neutralized *C. caviae in vitro* demonstrating an important contribution of humoral immunity in antigen-specific anti-*Chlamydia* responses. These observations are in accordance with other studies on the generation of antigen-specific humoral responses and IgG production [58], [60], [61] and the ability to neutralize *Chlamydia* [60].

Importantly, the novel guinea pig qRT-PCR transcriptome array revealed significant modulation in several innate immunity markers particularly associated with NK cells and Th1/Th2 specific cytokines and chemokines in immunized guinea pigs. Increased NK cell activation markers such as CD94, CD233 and IL-21 with concomitant downregulation of Th2/humoral responses and increases in Th1 responses were observed using the gene array (Fig. 5, Tables 1, 2). NK cells have been reported to contribute to IFN-γ production following genital *Chlamydia* infection leading to the development of Th1-CD4^+^ T cell responses and subsequent clearance of infection [62]. When comparing *C. caviae* EB vaccinated animals with mock vaccinated guinea pigs, increased Th1 and Th2 gene-related responses were observed at day 3, with Th2 responses remaining enhanced at day 9 post challenge. The robust cellular responses indicated by the gene expression array correlated with partial bacterial clearance by day 3 and complete clearance by day 9 post chlamydial challenge (Fig. 6). Additionally, RANTES, a major T cell chemokine was markedly upregulated 9 days post challenge in mock vaccinated guinea pigs but not in vaccinated animals; consistent with the kinetics of bacterial clearance from the infected genital tract (Fig. 6). RANTES gene regulation has been reported in a male guinea pig genital chlamydial infection model, with elevated levels of this chemokine associated with T-cell influx into the urethra following *C. caviae* challenge [63]. Additionally, as reported by Sakthivel *et.al*. [64], inhibition of RANTES (CCL5) in mice led to reduced antigen-specific activation (IL-12 and IFN-γ production) of CD4^+^ T cells isolated from lymphoid tissues and genital tract, and an associated prolonged *C. muridarum* shedding.

Other differentially expressed immune genes revealed by the array, including IFN-γ, CXCL10, IFNAR1 and OX40L, have been well documented to be associated with genital chlamydial infection in the murine model [65], [66], [67] but have not previously been examined in guinea pigs. Additionally, from the gene array, we observed modulation of genes not been previously reported in chlamydial infections. CD36 was up-regulated in the lower and upper genital tract on day 9. CD81 and CD130 were found to be up-regulated, and IL-21 and CD96 were down-regulated, in the upper genital tract on day 9. CD36 is expressed on monocytes/macrophages and has a critical role to play in atherosclerotic lesions [68]. CD81 is expressed on B cells, T cells and dendritic cells and has been shown to co-stimulate T cell activation and is required for induction of Th2 biased immune responses [69], [70] In contrast, the role of IL-21 (an NK and T cell activator; upregulated in non-protected guinea pigs, Table 1) has been implicated to be important in HIV induced CD8^+^ T cell activation and poorer disease outcomes [71], has not been previously investigated in *Chlamydia*-induced pathology. These data additionally make the guinea pig a useful model to study chlamydial pathogenesis.

Several guinea pig gene arrays that were created prior to completion of the genomic sequence and subsequent annotation have been used to study other bacterial infections [72], [73]. For example, a custom-made oligonucleotide (60 mer) microarray (81 spots) was used to analyze the mRNA expression of multiple cytokines and immune-related genes following *Mycobacterium* BCG vaccination [73]. In the study by Tree *et.al*. [73], 11 differentially expressed genes with greater than 1.4 fold change were identified in splenocytes re-stimulated with purified protein derivative of *M. tuberculosis*, indicating the induction of Th1-responses by BCG vaccination.

Although our immune gene specific-qRT-PCR array provides additional insights into the regulation of selected immune pathways following vaccination and *C. caviae* genital infection, we analyzed whole genital tract tissue, and thus, specific cell types responsible for particular gene expression were not identified. Subsequent studies using cell sorting techniques would be required to better appreciate the sources and locations of the differently expressed genes. Similarly, the multi-cell type nature of the tissues led to an averaging effect of the gene expression differences common to all methods of whole tissue analysis. Despite these limitations, these novel analyses provided an inclusive view of immune gene expression within the genital compartment of guinea pigs following vaccination and *C. caviae* challenge. These results extend our current understanding of the immune responses in this model of chlamydial infection of the genital tract and extend the utility of this animal model for the study of chlamydial pathogenesis.

## Supporting Information

S1 Figure Effect of *C. caviae* EB Vaccination on Histopathology in the Oviduct and Cervix from Guinea Pigs following Chlamydial Challenge. The genital tracts of each guinea pig were removed at day 80 post-challenge with *Chlamydia*, sectioned and analyzed using microscopy following H&E staining. Injury scores were calculated using the criteria as described in the Methods. Graphs are expressed as mean ± SD and compared using paired t-test. The asterisk indicates statistically significant differences (* *p*<0.05) between *C. caviae* vaccination and mock vaccination group for the edema score.
(TIF)

S2 Figure Illustration of the Guinea Pig Immune Gene Array.
(TIF)
