# Supplementary figures and images for: Use of a Guinea Pig-Specific Transcriptome Array for Evaluation of Protective Immunity against Genital Chlamydial Infection following Intranasal Vaccination in Guinea Pigs
Source: PLoS One. 2014 Dec 11;9(12):e114261. doi: 10.1371/journal.pone.0114261 (PMC4263467; doi:10.1371/journal.pone.0114261)

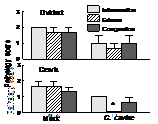

Supplement: S1 Figure — Effect of C. caviae EB Vaccination on Histopathology in the Oviduct and Cervix from Guinea Pigs following Chlamydial Challenge. The genital tracts of each guinea pig were removed at day 80 post-challenge with Chlamydia, sectioned and analyzed using microscopy following H&E staining. Injury scores were calculated using the criteria as described in the Methods. Graphs are expressed as mean ± SD and compared using paired t-test. The asterisk indicates statistically significant differences (* p<0.05) between C. caviae vaccination and mock vaccination group for the edema score. (TIF) [file pone.0114261.s001.tif]
